# Current-driven coherent skyrmion generation

**DOI:** 10.1038/s41598-019-40220-6

**Published:** 2019-03-05

**Authors:** C. Deger, I. Yavuz, F. Yildiz

**Affiliations:** 1 Marmara University, Physics Department, 34722 Ziverbey, Istanbul Turkey; 2 Gebze Technical University, Department of Physics, 41400 Kocaeli, Turkey

## Abstract

The next-generation logic and memory devices using magnetic skyrmions as spintronic information carriers are frequently studied, thanks to their remarkable magnetic stability, extremely compact size and very-low-cost driving forces within nanotracks. In order to realize skyrmion-based spintronic devices, understanding the skyrmion generation and their dynamics are essential. In this study, we have carried out a systematic micromagnetic simulation study on coherent magnetic skyrmion generation in which we theoretically engineered nanotracks by embedding an anti-notch to a channel of certain width. We found that the drift velocity and the skyrmion generation frequency can be tailored by the applied spin-polarized DC current density. Moreover, skyrmion generation is crucially affected by both damping and nonadiabaticity parameters, as well as the geometry of the anti-notch. We anticipate that our predictions provide rational basis for skyrmion-based devices in which skyrmions are used as information carriers, and influence future discussions.

## Introduction

Magnetic skyrmions are micron or sub-micron sized particle-like magnetic configurations which have magnetization antiparallel to magnetic field at their centre and parallel to the field at their periphery^1,2^. The potential and diversity of future skyrmionic devices have attracted the interest of numerous investigators. Especially, the next-generation logic and memory devices, which can fundamentally use the skyrmions as the information carrier, are frequently investigated due to its remarkable magnetic stability, extremely compact size and very-low-cost driving force^3–11^. The first observations of Bloch-type magnetic skyrmions have been achieved in B20 materials having a non-centrosymmetric crystalline structure. However, the formation of the skyrmions in B20 materials had required certain conditions, such as low ambient temperature and external magnetic field^4,12–14^. Later, the attention focused on the ferromagnetic/non-magnetic interfaces having a broken inversion symmetry, which can induce strong Dzyaloshinskii-Moriya interaction (DMI)^15,16^. Even though the formation of Néel-type skyrmions was achieved at the interfaces exhibit strong DMI, the challenges in the creation of skyrmions were remained, *i*.*e*., low temperature conditions and high external magnetic field are still required^17–19^. Finally, Pt/Co/Ta and Co/Pd multilayers with perpendicular magnetic anisotropy (PMA) allowed the first observation of room temperature zero field skyrmions^3,5,14^. Further, these systems are considered to be the most suitable candidates for industrial applications since they allow large spin currents resulting in faster skyrmion motions^14^.

In addition to the stability of the skyrmions, the generation and their motion along nanostructures must be accomplished to use skyrmions as efficient information carriers. However, topological stability of skyrmions restrains the creation (or annihilation) by a continuous variation in spin configuration from a uniform ferromagnetic state^20^. Fortunately, the creation of single skyrmion has been achieved by circulating current^21^, ultra-short single optical laser pulses^22^ and sub-nanosecond spin-orbit torque pulses^23^. Continuous multiple skyrmion generation has recently been demonstrated by Ma *et al*. via pulsations of microwave field or spin-polarized current density^24^. Moreover, the demonstration of current-driven transformation from stripe domains to magnetic skyrmion bubbles was recently performed in Ta/CoFeB/TaO trilayers^25^.

**Figure 1:**
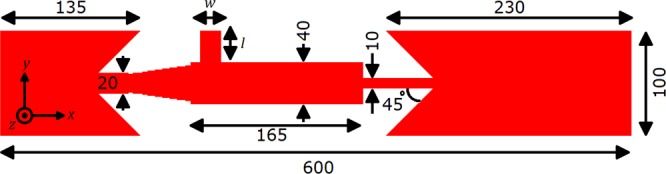
The design of the magnetic skyrmion generator. We inject the spin-polarized current into the nanowire with current-in-plane (CIP) geometry. The electrons flow toward +*x* direction, *i*.*e*. the current flows toward −*x* direction. The current density changes proportionally by the width of the wide or narrow parts of the nanowire. Thus, the current density inside the right chamber is equal to that inside the left chamber. *w* and *l* represent the width and length of the anti-notch, respectively. Here, the units are in nm.

In this work, using micromagnetic simulations, we propose a novel design for coherent field-free generation of multiple skyrmions, which can efficiently supply continuous logic information in a race-track memory device, with no time-dependent stimulation. This is accomplished by purposely implementing an anti-notch to a nanotrack as shown in Fig. 1. The spin transfer-induced motion of skyrmions and domain walls in magnetic tracks with an anti-notch is extensively investigated. The spin-polarized DC current-induced skyrmion and domain wall dynamics is found to be strongly dependent on the expanded geometry and boundary effects. We anticipate that our study not only offers a rational geometry for coherent skyrmion generation but also will inspire future considerations about the influence of the enlargement of the nanotracks on current-driven motion of the skyrmions and domain walls.

## Micromagnetic Framework

The theoretical realization of the proposed coherent skyrmion generation is made possible by micromagnetic simulations based on the Landau-Lifshitz-Gilbert (LLG) equation. The simulations are performed using the software Mumax3^26^. The analytic form of the LLG equation, considering separately spin torques Γ_*ST*_ associated with the current flowing in the film plane (CIP), can be written as^27–29^

1 $$\frac{\partial \overrightarrow{m}}{\partial t}=-\,\gamma \overrightarrow{m}\times {\overrightarrow{H}}_{eff}+\alpha \overrightarrow{m}\times \frac{\partial \overrightarrow{m}}{\partial t}+{\overrightarrow{{\rm{\Gamma }}}}_{ST},$$

where *γ* and *α* are, respectively, the gyromagnetic ratio and the Gilbert damping constant. The first and second terms of the right-hand side of Eq. (1) are the precession and damping terms. We modified the first term and inserted an additional term to the LLG equation, which are Dzyaloshinskii-Moriya interaction term and spin torque term, respectively. The DMI term is included in the system by embedding it in the effective field term (*H*_*eff*_) of the LLG equation. The Dzyaloshinskii-Moriya energy between two interacting sites is given by^16,30–32^

2 $${E}_{ij}={\overrightarrow{D}}_{ij}\cdot ({\overrightarrow{S}}_{i}\times {\overrightarrow{S}}_{j}),$$

where ${\overrightarrow{D}}_{ij}$ is the DMI vector which is given by $d{\hat{u}}_{ij}\times \hat{z}$^17,33–36^, where ${\hat{u}}_{ij}$ is the unit vector between sites i and j. Here, $\hat{z}$ represents the unit vector perpendicular to the film plane. We assume a uniform average value along the film thickness by defining a magnetization direction $m(\overrightarrow{r})$ at position $\overrightarrow{r}$, even though DMI is originated at the interfaces. Thus, the energy originated by DMI, in units of J/m^2^, is represented by^30,37,38^

3 $${E}_{DM}=t\int \int D[({m}_{x}{\partial }_{x}{m}_{z}-{m}_{z}{\partial }_{x}{m}_{x})+({m}_{y}{\partial }_{y}{m}_{z}-{m}_{z}{\partial }_{y}{m}_{y})]{d}^{2}\overrightarrow{r},$$

here, *D* is the continuous effective DM interaction constant across the thin-film.

The other additive term of the LLG equation, spin torque, ${\overrightarrow{{\rm{\Gamma }}}}_{ST}$, associated with current flowing in the film plane is expressed in the Zhang-Li form^26,27,29^

4 $${\overrightarrow{{\rm{\Gamma }}}}_{st,CIP}=-\,({\overrightarrow{v}}_{s}\cdot \overrightarrow{\nabla })\overrightarrow{m}+\beta \overrightarrow{m}\times ({\overrightarrow{v}}_{s}\cdot \overrightarrow{\nabla })\overrightarrow{m}$$

where ${\overrightarrow{v}}_{s}$ is effective spin-current drift velocity. The magnitude of ${\overrightarrow{v}}_{s}$ can be written in an explicit form as^27^

5 $$|{\overrightarrow{v}}_{s}|=-\,{\mu }_{B}p/[e{M}_{s}\mathrm{(1}+{\beta }^{2})]j$$

where *μ*_*b*_, *j* and *β* are Bohr magneton, current density and nonadiabatic spin torque parameter, respectively.

For micromagnetic simulations, 1-nm-thick cobalt nanotracks with length of 600 nm and width of 10∼100 nm on the substrate is considered. The geometry can be seen in Fig. 1. The following parameters are adopted to represent intrinsic properties of the layer^1,5,7^: M_*s*_ = 580 kA/m (saturation magnetization), A = 15 pJ/m (exchange stiffness), D = 3.5 mJ/m^2^ (DMI constant), *α* = 0.3 (Gilbert damping coefficient) and K_1_ = 0.8 MJ/m^3^, where K_1_ represents the constant of the-first order uniaxial magnetic anisotropy, which is an effective anisotropy includes contributions from the surface and shape anisotropy. The shape anisotropy originated from the dipole-dipole interactions causes a shift in the constant of uniaxial anisotropy in ultra-thin film cases where the non-local effects are negligible^39,40^. Our samples are discretized into 600 × 150 × 1 unit cells, corresponding to cell size of 1 × 1 × 1 nm^3^, which is smaller than both the  Néel exchange length ${\lambda }_{Neel}=\sqrt{2A/({\mu }_{0}{M}_{S}^{2})}\mathrm{=8.42}$ nm and the Bloch exchange length ${\lambda }_{Bloch}=\sqrt{A/K}=4.33$ nm.

The detailed geometry of the nanotrack is as follows: it consists of three chambers (left, right and central chambers) and are connected by passages. The anti-notch having variable width (*w*) and length (*l*) is placed on the left-most section of the central passage. The geometrical parameters of the nanotrack is shown in Fig. 1. For our micromagnetic simulations, we first created a skyrmion at the center of left chamber of the nanotrack. Next, we allow the system to  relax to a current-free energy minimum state. Then the timer is started and we inject the spin current with a polarization (P) of 1.0 into the nanotrack in the CIP geometry. Subsequently, the current flows toward the left direction so the electrons would flow toward the right. Unless otherwise specified, the non-adiabatic torque coefficient (*β*) is chosen as to be equal to the damping coefficient, *i*.*e*. 0.3, which provides stabilization for the motion of skyrmions along a straight horizontal line through the nanotrack without an additional traverse force.

## Results and Discussions

### Generation process of skyrmions

**Figure 2:**
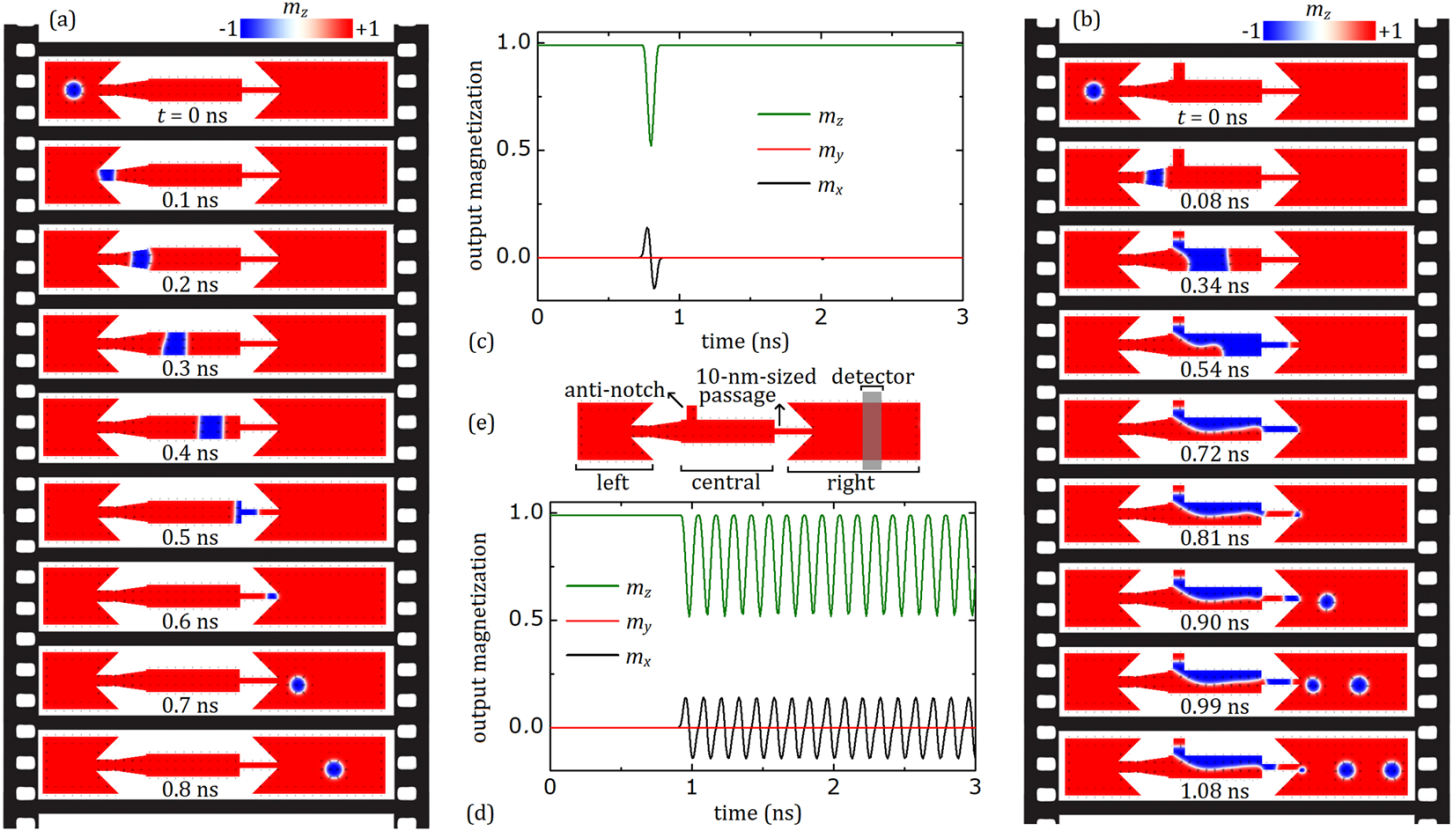
Reversible conversion between skyrmion-DW pair and coherent skyrmion generation. (**a**) A skyrmion is created in the left chamber of the nanotrack. Then, it is drifted by current to the central chamber without the anti-notch, by transforming into a DW pair. Finally, it is, again, converted to the skyrmion by the passage in the entrance of right chamber. A current density of 7 TAm^−2^ is applied along −*x* for 3 ns. However, the spatial magnetization is represented as frames for 0 ns < *t* < 0.8 ns to show the conversion process in more detail. (**b**) The coherent skyrmion generation after introducing the central chamber to the anti-notch with the dimensions of *w* = 20 nm, *l* = 30 nm. The current density is again 7 TAm^−2^. Selected frames of the skyrmion generation process is represented from *t* = 0 ns to *t* = 1.08 ns. (**c**) x, y and z components of the total magnetization in the detector area (output magnetization) with respect to simulation time in the absence of anti-notch. (**d**) The output magnetization with respect to simulation time in the presence of anti-notch. (**e**) Skyrmion generator with the detector. The detector has the width of 30 nm and length (height) of 100 nm and its center is located at 85 nm far from the end of the right chamber.

To realize the proposed generation process, the conversion between a skyrmion and a domain-wall (DW) pair must be well-understood. Magnetic thin film multilayers with perpendicular magnetic anisotropy can comprise strong DMI at the interfaces, which consist of a heavy metal layer with high-spin-orbit coupling and a magnetic layer. These multilayers could allow the stabilization of chiral DWs and/or skyrmions^25^. A nanowire geometry which leads to the reversible conversion between a skyrmion and a domain-wall pair is considered in Fig. 2(a). In this case, skyrmion was first created in the left chamber with an intrinsic diameter of 30 nm, which is determined by the material parameters and confined geometry at *t* = 0. The created skyrmion survives until it is drifted by the applied current toward the end of left chamber having a narrow passage with a width of 20 nm. This narrow passage, normally, does not allow the stabilization of a skyrmion with such diameter, *i*.*e*. larger than the width of the passage^41^. Therefore, the skyrmion is transformed into a DW pair at *t* = 0.1 ns. The DW pair propagates rightwards in the central narrow chamber until the DW reaches a 10-nm-sized passage, as shown in Fig. 2(a) and at *t* = 0.5 ns. Then, DW pair is converted into a skyrmion until *t* = 0.7 ns. This exotic behavior is both theoretically proposed^41^ and experimentally observed^25^ recently.

**Figure 3:**
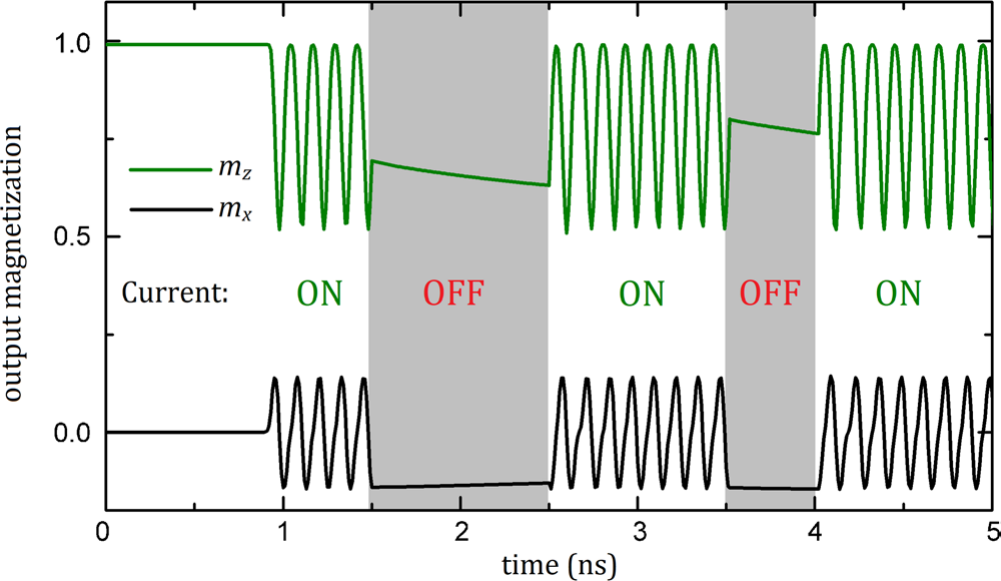
Sustainability of multiple skyrmion generation. The response of multiple skyrmion generation to switching of the current at random moments. The current density, *j*, of 7.0 TAm^−2^ is applied when the current is switched on. The anti-notch dimensions of *w* = 20 nm and *l* = 30 nm is employed.

The story is a lot different when an anti-notch introduced to the central chamber, as can be seen in Fig. 2(b). The current-driven skyrmion again moves to the rightward direction and is converted into a DW pair until *t* = 0.08 ns. Once the DW pair reaches to the anti-notch, one side of the DW pair is pinning inside the anti-notch while the other side continues to flow toward the end of the central chamber, causing an expansion in the domain dimensions until *t* = 0.54 ns. Addition of an anti-notch to the wire also results in an inhomogeneity in the current flow; *i*.*e*. an additional current component along the y axis. Inhomogeneous effective forces on the DWs caused by inhomogeneous current act to narrow the domain around the entrance of the 10-nm-sized passage, which is located at the end of the central chamber. This narrowing results in the breaking of the stripe at *t* = 0.72 ns. The broken part (remained at the end of central chamber) of the DW propagates to the entrance of the 10-nm-sized passage until *t* = 0.81 ns, at the same time, the clipping part of the stripe starts to blow a magnetic skyrmion bubble. Inhomogeneous effective forces, this time, act to expand the end of the domain in the entrance of the right chamber of the nanowire. As the domain end expands its radius, the surface tension in the DW (originated from the increasing in the energy of DW comprises the combination of exchange and anisotropy fields) increases^25,42^, resulting in the formation of the skyrmion until *t* = 0.90 ns. This process resembles how liquid droplets are formed by leaking tap, which allow us to call these skyrmions as “skyrmion droplets”. The periodic repetition of the process enables the coherent generation of the skyrmions. We embedded a fictitious detector on the right chamber of the nanotrack, as can be seen in Fig. 2(e), to reveal the time evolution of the skyrmion generation, especially the frequency. The detector measures the x, y and z components of the total normalized magnetization during simulations, lasting for 3 ns, and will be called as output magnetization. In Fig. 2(c), the peaks of *m*_*x*_ and *m*_*z*_ corresponds to signal of skyrmion passage through the detector in the central chamber without an anti-notch (see Fig. 2(a)) around *t* = 0.8 ns. No skyrmion propagation is observed after *t* = 0.8 ns, which indicates no multiple skyrmion generation in the absence of anti-notch. Figure 2(d) represents the output magnetization of anti-notch added geometry. It can be seen that multiple skyrmion generation in the right chamber is stable and coherent during the simulation. To investigate the response of multiple skyrmion generation to alternating current, we calculate the time dependence of output magnetization by switching the applied current at random instants during the simulation. The results are represented in Fig. 3. We found that the multiple skyrmion generation is sustainable with the switched current, which is obviously important for magnetic data storage applications. For the selected parameters, the generation frequency is 8.33 GHz. The frequency can be altered by both the intrinsic parameters of the material, *i*.*e*. the ratio of damping and non-adiabatic torque coefficients, and applied current.

### Current density - Generation frequency relationship

**Figure 4:**
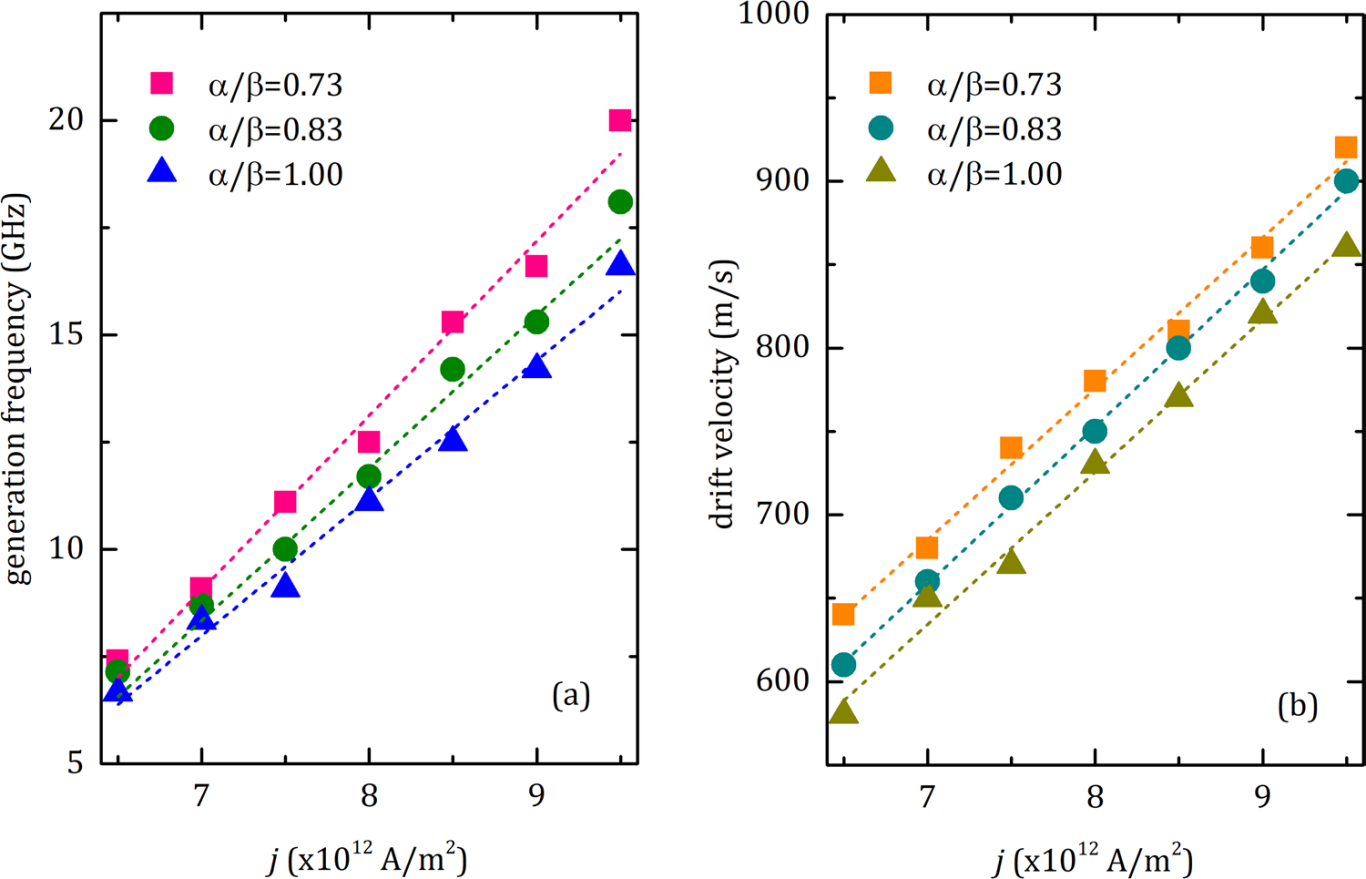
Skyrmion generation with respect to current. (**a**) The dependency of number of skyrmions generated per second (generation frequency, *f*) on current density, *j*, for the anti-notch dimension of *w* = 20 nm and *l* = 30 nm. We changed *j* from 6.5 TAm^−2^ to 9.5 TAm^−2^ with the steps of 0.5 TAm^−2^ for various *α*/*β* ratios. *β* is remained at 0.3 while *α* is varied to get desired *α*/*β* ratio. (**b**) Drift velocity of skyrmions as a function of current density. Dashed lines in each figure are the linear fits of the corresponding data. The linear fits in (**b**) are used to extract the mobility (velocity per current density) of the skyrmions.

Here, we study the impact of current density and *α*/*β* ratio on the dynamics of coherent skyrmion generation. We quantify the generation frequency, *f*, and drift velocity, ${\overrightarrow{v}}_{s}$, of the skyrmions. We scanned *f* and *v*_*s*_ as a function of current density *j* for three values of *α*/*β*. The results are shown in Fig. 4(a and b), respectively. For all values of *α*/*β*, we find a linear relationship between *j* and *f*, ${\overrightarrow{v}}_{s}$, that is, larger current density induces relatively high generation frequency and drift velocity. We find a threshold current density of 6.5 TAm^−2^, i.e. the minimum current density (*j*_*m*_) required to blow a skyrmion from the 10-nm-sized passage. It can also be seen that, smaller values of *α*/*β* result in higher generation frequency and drift velocity since lower damping in the system allows faster motion of skyrmions. By applying *j*_*m*_ for *α*/*β* = 1, we can generate skyrmions with drift velocity of 580 m/s corresponding to a reading speed of 6.4 Gb/s for a single race-track memory, given that the separation between each skyrmion is ∼90 nm. The reading speed can be enhanced up to 18.4 Gb/s for a single track with a skyrmion separation distance of ∼50 nm, by applying current density of 9.5 TAm^−2^, for *α*/*β* = 0.73. Using simultaneous multiple race-tracks can also increase the reading speed. The current-driven mobility of the skyrmions^43^, *μ* = *v*/*j*, was found to be roughly 92 ms^−1^/TA m^−2^, which is nearly independent of *α*/*β*.

### The role of anti-notch dimension

**Figure 5:**
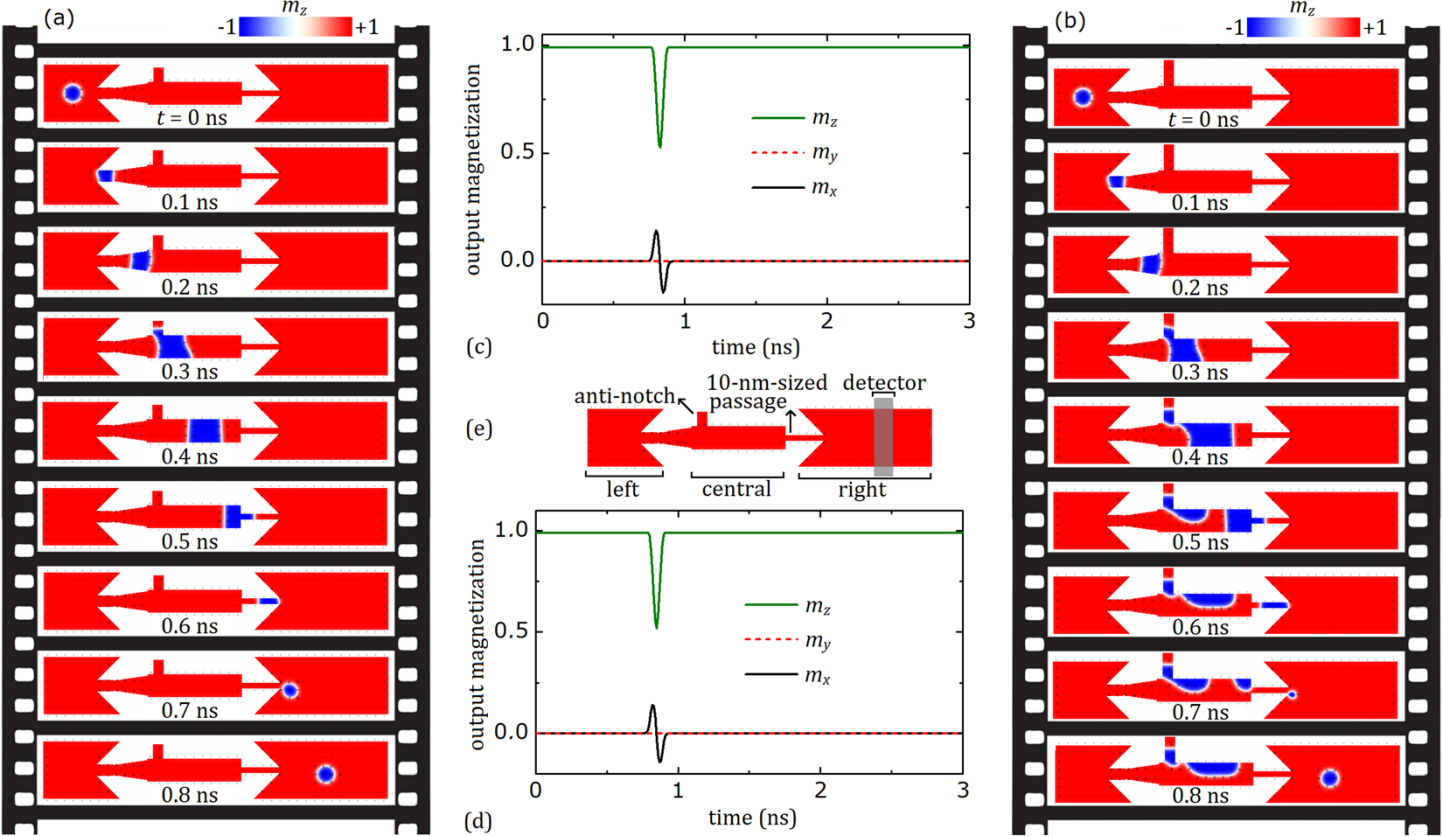
Two different anti-notch which prevent coherent skyrmion generation. The central chamber is introduced to an anti-notch with the dimensions of *w* = 18 nm, *l* = 25 nm in (**a**) and *w* = 18 nm, *l* = 45 nm in (**b**). A current density of 7 TAm^−2^ is applied along −*x* for 3 ns. The spatial magnetization is represented as frames for 0 ns < *t* < 0.8 ns to show the role of anti-notch dimensions on generation process in more detail. The output magnetizations with respect to simulation time in the presence of short and long anti-notch is represented in (**c**) and (**d**), respectively. (**e**) The nanotrack used for the simulations with the detector introduced in Fig. 2(e).

Even though we find that the embedding of the anti-notch results in the coherent skyrmion generation, there must be limitations for certain anti-notch width and length. Inserting the anti-notch to the central chamber gives rise to inhomogeneity in the current flow which corresponds to an additional current component along the y axis. The magnitude of the y-component of the current is strongly correlated with the aspect ratio of the anti-notch. Moreover, the pinning of the backside of the DW pair depends on the magnitude of the y-component. To investigate the cases of which anti-notch dimensions disrupt multiple skyrmion generation, we performed simulations for anti-notches having two different dimensions, as shown in Fig. 5. The width of the anti-notch remains constant while the length is determined as 25 nm and 45 nm in Fig. 5(a,b), respectively. In this case, the anti-notch in (a) and that of in (b) will be called as short and long anti-notch. The short anti-notch induces inadequate current flows toward the anti-notch, followed by passing of DW pair without pinning. Consequently, the coherent generation process does not occur, as can be seen in Fig. 5(c). The anti-notch with redundant length can also prevent the generation. In Fig. 5(b), the long anti-notch is embedded to the central chamber and current is applied. We noticed that long anti-notch induces large magnitude of y-component in current, which splits the DW pair around the anti-notch. In this case, residual part of the DW leaks in the anti-notch while the other part flows toward the right chamber and creates a single skyrmion. Even though the residual part in the anti-notch can create magnetic domains with the opposite direction of the magnetization of the nanowire, the domains can not reach the 10-nm-sized passage by vanishing at the upper-right edge of the central chamber. Figure 5(d) represents the time evolution of an unsuccessful coherent skyrmion generation by a long anti-notch.

**Figure 6:**
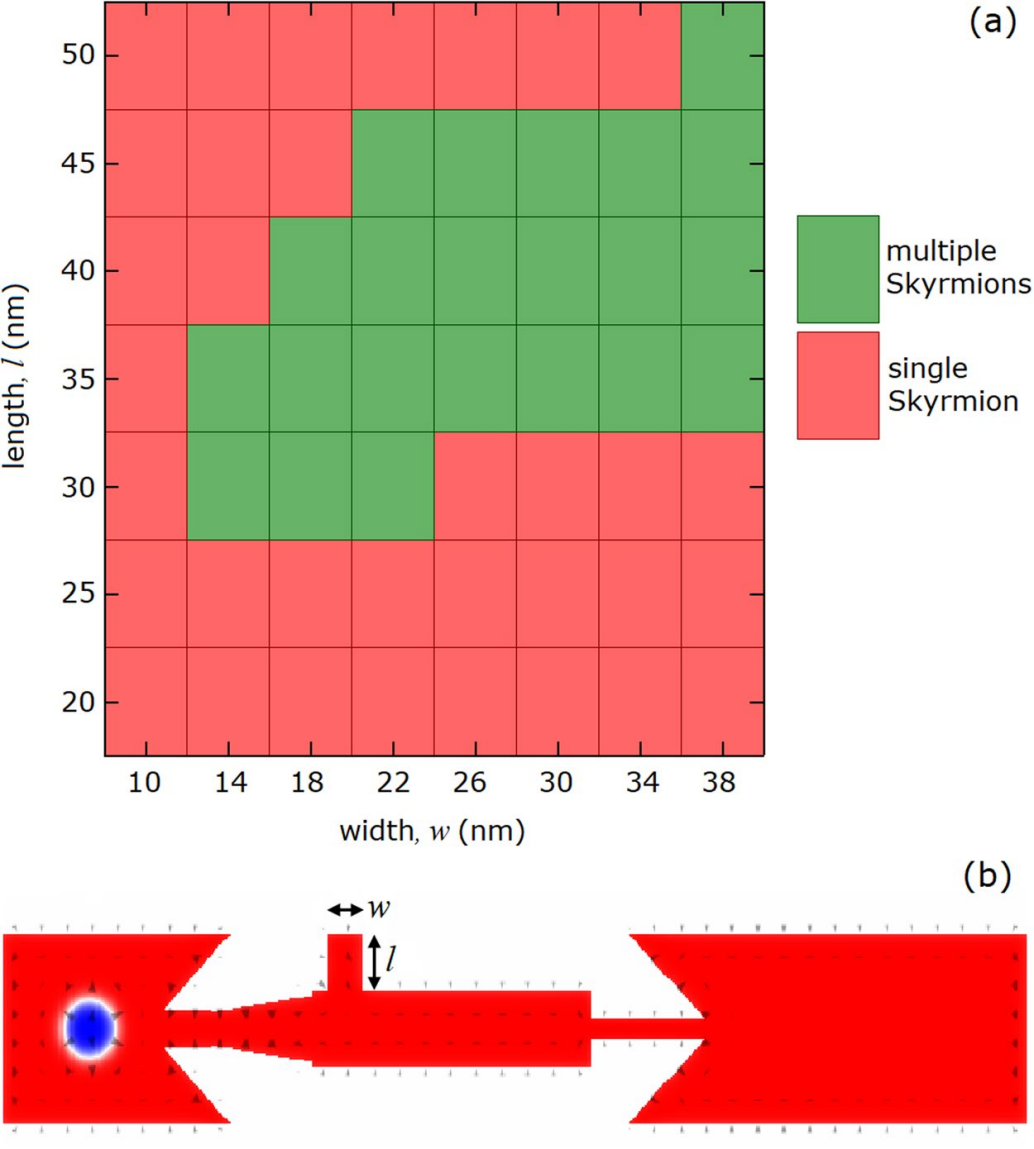
Dependency of coherent generation process on width and length of the anti-notch. (**a**) The effect of width (*w*) and length (*l*) on skyrmion generation. In the matrix, *w* and *l* values allowing coherent generation of skyrmions are represented by green boxes whereas the anti-notch geometries unfavorable for the generation appears as red. (**b**) The schematic representation of the nanotrack with anti-notch.

We compiled the role of width and length of the anti-notch by performing further analysis to represent the full picture, as shown in Fig. 6. We investigated the control of coherent skyrmion generation as a function of both width (*w*) and length (*l*) of the anti-notch. Green boxes correspond to the *w* and *l* values enabling a coherent generation of skyrmions while red boxes represent the anti-notch geometry preventing the multiple skyrmion generation, *i*.*e*. the output is a single skyrmion. It seems that there is a lower limit for *l* in multiple skyrmion generation, which corresponds to the skyrmion diameter. For lengths smaller than the skyrmion diameter, the DW pair passes through the central chamber without pinning. For *l* = 30 nm, we can observe all of the three scenarios: (1) the splitting of the DW pair followed by no coherent skyrmion generation for *w* = 10 nm, (2) coherent skyrmion generation by the optimum aspect ratios for *w* = 14–22 nm, (3) passing of the DW pair without pinning, again, followed by no coherent skyrmion generation for *w* > 22 nm. For larger values of *l* = 30 nm, the generation process becomes less selective since the only unfavorable scenario is the splitting of the DW pair by excessively long anti-notches. Additionally, the working conditions of successful coherent skyrmion generation for various material parameters are given in supplementary materials.

## Conclusion

In this study, we demonstrated a new road map for permanent current-driven coherent generation of magnetic skyrmions in the GHz frequency range, by embedding an engineered anti-notch to a nanotrack. We found that the anti-notch results in an inhomogeneity in the current flow which leads an electrical skyrmion oscillator producing a periodic chain of magnetic skyrmions without any periodic external influence. This procedure can be realized as an alternative method to the field and/or spin-polarized current pulsation in multiple skyrmion generation. The dynamics of current-driven skyrmion and DWs are extensively investigated as a function of the geometry of anti-notch and current density by micromagnetic simulations. The frequency of the generation and drift velocity of the skyrmions are also tailored by the applied current density. We found that the reading speed based on the generation can be enhanced up to 18.4 Gb/s for a single race-track, which can be increased by using multiple tracks.

## Supplementary information

Supplementary Info

Long_anti-notch

No_anti-notch

Optimum_anti-notch

Short_anti-notch
