# Supplementary material for: Current-driven coherent skyrmion generation
Source: Sci Rep. 2019 Mar 5;9:3513. doi: 10.1038/s41598-019-40220-6 (PMC6401069; doi:10.1038/s41598-019-40220-6)

Supporting Information

Current-driven coherent skyrmion generation

C. Deger[1], I. Yavuz[1*] and F. Yildiz[2]

[1] Marmara University, Physics Department, 34722, Ziverbey, Istanbul, Turkey

[2] Gebze Technical University, Department of Physics, 41400, Kocaeli, Turkey.

***corresponding author:**

e-mail: ilhan.yavuz@marmara.edu.tr

Contents:

Figure S1. Working conditions for successful coherent skyrmion generation for various uniaxial magnetic anisotropy and exchange stiffness values.

Figure S1. Working conditions for successful coherent skyrmion generation for various uniaxial magnetic anisotropy and exchange stiffness values.

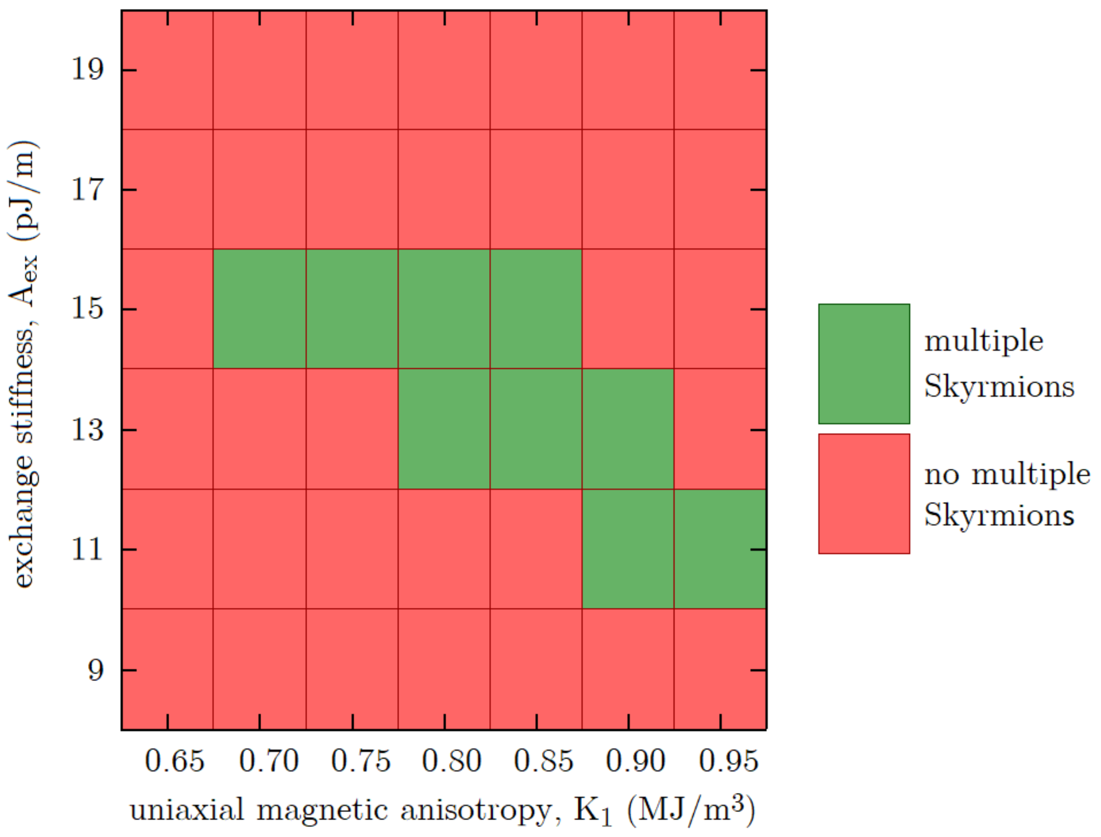

Supplement: Supplementary file 1 — Supplementary Info [file 41598_2019_40220_MOESM1_ESM.pdf]
