# Supplementary figures and images for: Current-driven coherent skyrmion generation
Source: Sci Rep. 2019 Mar 5;9:3513. doi: 10.1038/s41598-019-40220-6 (PMC6401069; doi:10.1038/s41598-019-40220-6)

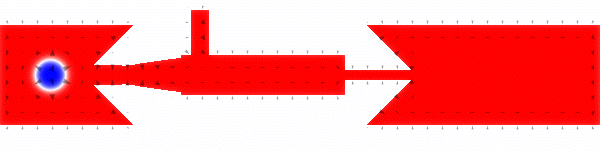

Supplement: Supplementary file 2 — Long_anti-notch [file 41598_2019_40220_MOESM2_ESM.gif]

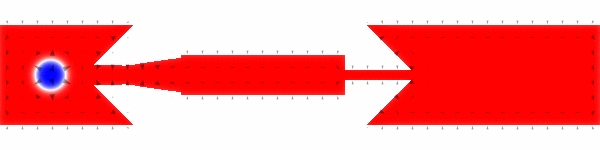

Supplement: Supplementary file 3 — No_anti-notch [file 41598_2019_40220_MOESM3_ESM.gif]

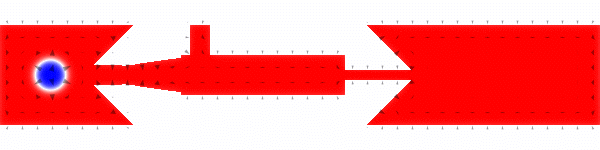

Supplement: Supplementary file 4 — Optimum_anti-notch [file 41598_2019_40220_MOESM4_ESM.gif]

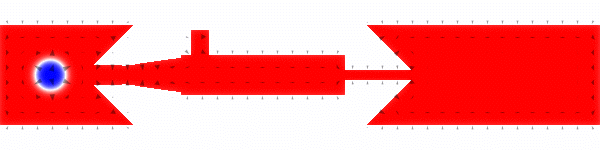

Supplement: Supplementary file 5 — Short_anti-notch [file 41598_2019_40220_MOESM5_ESM.gif]
